# Single nucleotide polymorphisms in the angiogenic and lymphangiogenic pathways are associated with lymphedema caused by *Wuchereria bancrofti*

**DOI:** 10.1186/s40246-017-0121-7

**Published:** 2017-11-09

**Authors:** Linda Batsa Debrah, Anna Albers, Alexander Yaw Debrah, Felix F. Brockschmidt, Tim Becker, Christine Herold, Andrea Hofmann, Jubin Osei-Mensah, Yusif Mubarik, Holger Fröhlich, Achim Hoerauf, Kenneth Pfarr

**Affiliations:** 1 Kumasi Centre for Collaborative Research in Tropical Medicine, Kumasi, Ghana; 2 Department of Clinical Microbiology, Kwame Nkrumah University of Science and Technology, Kumasi, Ghana; 3 Institute for Medical Microbiology, Immunology and Parasitology, University Hospital Bonn, Sigmund-Freud-Str. 25, 53127 Bonn, Germany; 4 Faculty of Allied Health Sciences of Kwame Nkrumah University of Science and Technology, Kumasi, Ghana; 5 Institute of Human Genetics, University of Bonn, Bonn, Germany; 6 Department of Genomics, Life and Brain Center, University of Bonn, Bonn, Germany; 7 Institute for Medical Biometry, Informatics and Epidemiology, University of Bonn, Bonn, Germany; 8 Bonn-Aachen International Center for Information Technology (B-IT), University of Bonn, Bonn, Germany

**Keywords:** Lymphatic filariasis, Angiogenesis, Lymphangiogenesis, Single nucleotide polymorphisms, Genotypes

## Abstract

**Background:**

Lymphedema (LE) is a chronic clinical manifestation of filarial nematode infections characterized by lymphatic dysfunction and subsequent accumulation of protein-rich fluid in the interstitial space—lymphatic filariasis. A number of studies have identified single nucleotide polymorphisms (SNPs) associated with primary and secondary LE. To assess SNPs associated with LE caused by lymphatic filariasis, a cross-sectional study of unrelated Ghanaian volunteers was designed to genotype SNPs in 285 LE patients as cases and 682 infected patients without pathology as controls. One hundred thirty-one SNPs in 64 genes were genotyped. The genes were selected based on their roles in inflammatory processes, angiogenesis/lymphangiogenesis, and cell differentiation during tumorigenesis.

**Results:**

Genetic associations with nominal significance were identified for five SNPs in three genes: vascular endothelial growth factor receptor-3 (VEGFR-3) rs75614493, two SNPs in matrix metalloprotease-2 (MMP-2) rs1030868 and rs2241145, and two SNPs in carcinoembryonic antigen-related cell adhesion molecule-1 (CEACAM-1) rs8110904 and rs8111171. Pathway analysis revealed an interplay of genes in the angiogenic/lymphangiogenic pathways. Plasma levels of both MMP-2 and CEACAM-1 were significantly higher in LE cases compared to controls. Functional characterization of the associated SNPs identified genotype GG of CEACAM-1 as the variant influencing the expression of plasma concentration, a novel finding observed in this study.

**Conclusion:**

The SNP associations found in the MMP-2, CEACAM-1, and VEGFR-3 genes indicate that angiogenic/lymphangiogenic pathways are important in LE clinical development.

**Electronic supplementary material:**

The online version of this article (10.1186/s40246-017-0121-7) contains supplementary material, which is available to authorized users.

## Background

Worldwide, more than 850,000 people live in areas endemic for *Wuchereria bancrofti*, *Brugia malayi*, and *Brugia timori* filial nematodes that cause lymphatic filariasis, a disease of severe morbidity [1]. Lymphatic disease symptoms are characterized by a cascade of events that leads to lymphatic dysfunction with associated fibrosis [2]. Lymphedema (LE) and hydrocele are pathologies that can develop in *Wuchereria bancrofti* infected individuals. These clinical symptoms are usually preceded by dilated and tortious lymphatic vessels and scrotal lymphangiectasia [3, 4]. Of these two pathologies, LE is the most debilitating, affecting about 7% of the population in a lymphatic filariasis (LF) endemic community even though all individuals in the endemic area may be inoculated with the parasite and the majority (80%) may be infected [5, 6].

LE is a condition caused by the leakage of plasma from the arterial blood capillaries that is then trapped in the soft tissues as a result of the dysfunction of the lymphatic vessel that originates from the infection with the filarial parasites *Wuchereria bancrofti* or *Brugia* spp. [7]. The global burden of LE in 2000 was 14.84 million [8]. After 13 years of treatment with ivermectin and albendazole or diethylcarbamazine, to eliminate the infection [1], and morbidity management procedures, there still remained 14.41 million LE cases [8], although an estimated 116–250 million DALYS have been averted within that period. This highlights the need for alternative strategies to current morbidity management procedures to help prevent or even ameliorate LE in the affected persons.

Individuals infected with lymphatic filariasis parasites do not show recognizable clinical symptoms. However, a third of those infected developed a clinical disease. What causes the expression of clinical disease is not well understood. Several reasons have been given to explain the differences in the cause(s) of heterogeneity in infection and disease of filarial infection. These include the immune interaction between the human host and the parasite [9–12], transmission potential of the mosquito vector [13], in utero exposure to parasite antigens [14, 15], and secondary bacterial/fungal infections superimposed on the lymphatic dysfunction [16].

The contribution of host immunogenetics to this heterogeneity has also been investigated, leading to the finding that susceptibility to infection, parasite load and pathology cluster in families [17–21], indicating an underlying genetic component is involved in the disease. Gene polymorphisms such as the variant Leu10Pro of transforming growth factor-β-1 (TGFβ-1) was found to be associated with both lack of microfilariae and differential microfilarial loads [22]. In that study, it was shown that the differential microfilaria loads and the lack of circulating microfilariae (Mf) in the blood exhibited by people in endemic areas have genetic propensity. Hence, some people in endemic areas may be infected with the adult worm but would have no Mf in the peripheral blood. Also, polymorphisms in TLR-2 (+ 597 > C, 1450T > C and −96 to −173 deletion) were found to be associated with higher asymptomatic bancroftian filariasis [23]. Association has also been found in the HH variant of Chitinase-1 (CHIT-1) that correlated with decreased activity as well as levels of chitotriosidase and susceptibility of filarial infection. The XX genotype in the mannose-binding lectin-2 (MBL-2) genes has been associated with susceptibility to bancroftian infection [24]. Positive association was reported for all variants of rs733618 of cytotoxic T-lymphocyte-associated protein 4 (CTLA-4) gene among asymptomatic amicrofilaremic cases [25]. IL-10 promoter haplotypes and *IL-10 RA* S138G polymorphisms have also been identified as possible genetic determinants of susceptibility to lymphatic filariasis [26]. All the above SNPs that have been found to be associated with filarial infections were the basis for our study.

We were among the first to show that angiogenic/lymphangiogenic molecules such as vascular endothelial growth factors (VEGFs) may be involved in the development of LE and hydrocele in humans [27, 28]. In these studies, we showed that VEGF-C and its receptor VEGFR-3 are elevated in the plasma of LE patients and treating them with antiangiogenic drugs such as doxycycline reduced the factors prior to ameliorating early stages of pathology [27]. We went further to show that another angiogenic molecule, VEGF-A, is genetically associated with hydrocele caused by bancroftian infections. Treatment with doxycycline again reversed the pathology in men with early stages of hydrocele [28]. Other authors have also shown the involvement of angiogenic/lymphangiogenic molecules in the clinical manifestations of LF [29, 30].

SNPs in FOXC-2 and FLT-4 genes have been identified to be involved in lymphedema progression [31].

While LE is clinically well described, there have been few investigations of host genetic contributions to filarial LE. In this study, we have further shown an association of SNPs in genes of the angiogenic/lymphangiogenic pathways with LE. Identified SNPs could contribute to the search of biomarkers for diagnosis of LE and potential methods to ameliorate LE symptoms.

## Results

### Demographic and pathology information of study participants

The mean age of study participants was not statistically different between cases and controls (Table 1). Predominantly, 71% of them were females and 29% were males. In the control group, the majority were males (57%). The volunteers had stayed in the study community from a year to over 50 years. In the cases group, 171 people (60%) had been a resident for more than 40 years. A greater number of cases had stages 2 and 3 (32 and 37%, respectively) pathology according to Dreyer et al. [32, 33], while stages 4 and 7 (2% each) were the least frequent stage of pathology among the cases (Table 1).

**Table 1:** Demographic and pathology profile of study participants

Variable	Cases *N* = 285	Controls *N* = 682
Mean age/years (range)	44.4 (16–73)	40.8 (16–93)
Gender		
Male % (*N*)	29.5 (84)	57 (389)^a^
Female % (*N*)	70.5 (201)	43 (293)
Duration in community/years		
1–10	2	55
11–20	10	90
21–30	42	178
31–40	57	132
41–50	79	109
> 50	92	118
Stages of lymphedema		
Stage 1	11	–
Stage 2	90	–
Stage 3	106	–
Stage 4	5	–
Stage 5	20	–
Stage 6	49	–
Stage 7	4	–

^a^Fisher’s exact test, *P* ≤ 0.05 controls compared to cases

### Single marker analysis

One hundred and forty-seven (147) single nucleotide polymorphisms (SNPs) were initially selected for genotyping (Additional file 1). Sixteen (16) were rejected during the assay design because the primer sequences produced were prone to primer dimerization or the masses of the sequences were too similar to be distinguished by mass spectrometry. Eight out of the 16 rejected SNPs are in the coding region resulting in amino acid changes, three were in the promoter region with no amino acid change, and six were in non-coding regions (Additional file 1). With the exception of tumor necrosis factor-α (TNF-α), CTLA-4, and interleukin-4 (IL-4), all the rejected genes were represented by at least one other SNP in the Sequenom data. Thus, 131 SNPs in 64 genes were genotyped.

The single marker analysis compared 285 LE patients (cases) and 682 infected patients without LE pathology (controls). Of the 131 SNPs genotyped, 5 SNPs in three genes were associated with LE with nominal significance (Table 2): 2 SNPs in matrix metalloprotease-2 (MMP-2 rs1030868, *P* = 0.0094; rs2241145, *P* = 0.0116), 2 SNPs in carcinoembryonic antigen-related cell adhesion molecule-1 (CEACAM-1 rs8110904, *P* = 0.024; rs8111171, *P* = 0.026), and 1 SNP in vascular endothelial growth factor receptor-3 (VEGFR-3 rs75614493, *P* = 0.034). None of the nominally associated SNPs withstood correction for multiple testing (Benjamini-Hochberg). All the associated SNPs were in Hardy-Weinberg equilibrium (HWE, *P* > 0.05) with the exception of CEACAM-1 rs8110904 (controls *P* = 2.93E–10).

**Table 2:** Genotype frequencies and odds ratio of SNPs associated with lymphedema patients and infected controls

Gene (dbSNP rs#)	Functional category of SNP	Genotypes	Cases (%)	Controls (%)	*P* value		OR^c^ (95% CI)
					P_ATT_^a^	Adjusted^b^	
CEACAM-1 (rs8110904)	Missense	AA	26 (9)	57 (9)	0.024	0.370	(A) 1.2 (0.99–1.49)
		AG	182 (67)	385 (59)			
		GG	65 (24)	213 (32)			
CEACAM-1 (rs8111171)	Missense	GG	61 (22)	205 (31)	0.026	0.370	(T) 1.3 (1.03–1.56)
		GT	145 (52)	311 (46)			
		TT	73 (26)	157 (23)			
FLT-4/VEGFR-3 (rs75614493)	Missense	CC	282 (99)	658 (96)	0.034	0.232	(C) 3.4 (1.02–11.29)
		CT	3 (1)	24 (4)			
		TT	0 (0)	0 (0)			
MMP-2 (rs1030868)	Intron	AA	69 (24)	113 (17)	0.0094	0.232	(A) 1.3 (1.07–1.58)
		AG	134 (47)	337 (49)			
		GG	82 (29)	232 (34)			
MMP-2 (rs2241145)	Intron	CC	86 (30)	158 (23)	0.0116	0.232	(C) 1.3 (1.06–1.57)
		CG	136 (48)	334 (49)			
		GG	63 (22)	190 (28)			

^a^Cochrane-Armitage test for trend

^b^Adjusted *P* values according to Benjamini-Hochberg

^c^Odds ratio with 95% confidence intervals for the risk allele

The risk alleles for MMP-2 SNPs rs1030868 and rs2241145 were A and C, respectively, each conferring a 1.3-fold risk to LE development (Table 2). Both alleles fit in a recessive model of association (Additional file 2: Table S1). No individual in the cohort was homozygous for the T allele in VEGFR-3 SNP rs75614493, and only three people were heterozygous (Table 2). The participants with the C allele of the VEGFR-3 SNP had a 3.4-fold risk of LE development. Due to the lack of homozygosity for the T allele in the cohort, no model of association, whether dominant or recessive, could be assigned (Additional file 2: Table S1). The risk alleles for CEACAM-1 SNPs rs8110904 and rs8111171 were A and T and confer a 1.2- and 1.3-fold risk, respectively, of LE development (Table 2). Both alleles fit a dominant model of association (Additional file 2: Table S1).

### Haplotype analysis

Two or more SNPs in a gene or on the same chromosome can form haplotypes that are inherited together [34]. Analysis of haplotype association with LE was done using the FamHap software package [35]. A likelihood ratio test with one degree of freedom was used to assess the significance of haplotype frequencies among SNPs on the same gene. Only haplotypes that were significantly associated at one degree of freedom were reported (Table 3).

**Table 3:** SNP haplotypes in genes associated with lymphedema

Gene	dbSNPrs#	Allele	Distribution cases (%)	Distribution controls (%)	*P* value (1df)	Global *P* value^a^
CEACAM-1	rs8110904	G	48.3	54.0	*0.026*	0.092
	rs8111171	G				
	rs8110904	A	48.0	39	*0.046*	0.092
	rs8111171	T				
VEGFR-3	rs75614493	T	0.5	1.8	*0.023*	0.080
	rs3587489	T				
MMP-2	rs2241145	T	33.1	28.3	*0.046*	*0.030*
	rs1030868	A				
	rs11643630	C				
	rs1992116	G				

Italic text indicates a significant association

^a^Calculated by an omnibus statistic with 200,000 simulations in FamHap version 19

Two CEACAM-1 SNPs, rs8110904 and rs8111171, were associated with LE in a single marker analysis with nominal significance. From these SNPs, three haplotypes were generated. The frequency of haplotype GG was significantly higher in the controls than the cases (*P* = 0.026); there was a trend in haplotype AT (*P* = 0.055) but there was no difference in haplotype GT between cases and controls (Table 3).

Six different haplotypes comprising SNPs rs11643630, rs1030868, rs2241145, and rs1992116 in the MMP-2 gene (GACG, GGCA, GGGG, TACG, TGCG, and TGGG) were predicted by the FamHap analysis. Haplotype TACG was significantly higher in cases than controls (*P* = 0.046), and this significance was even strengthened after multiple testing with 200,000 simulations, (*P* = 0.03, Table 3). The remaining haplotypes were not significant in either cases or controls.

The VEGFR-3 SNPs rs75614493 and rs3587489 formed three haplotypes (CC, CT, and TT). The TT haplotype was rare in this population and was significantly associated with controls (*P* = 0.023), but was lost after correcting for multiple testing (*P* = 0.08, Table 3).

### Plasma levels of angiogenic/lymphangiogenic molecules

**Fig. 1:**
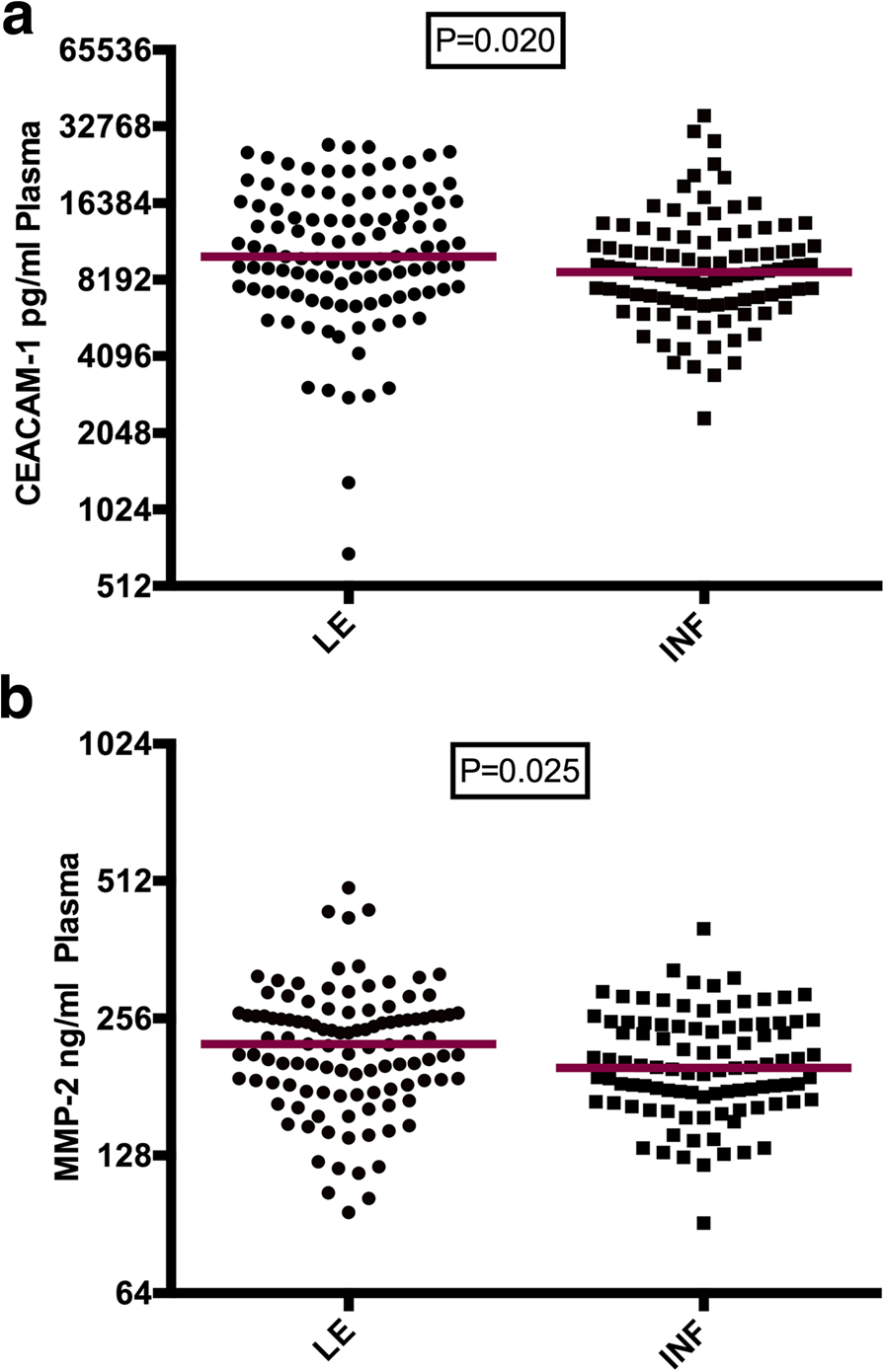
Plasma concentrations of CEACAM-1 and MMP-2 are higher in lymphedema patients. **a** Plasma concentration of CEACAM-1 gene. **b** Plasma concentration of MMP-2 gene. EDTA Plasma was collected from 101 LE patients and 99 infected patients without disease symptoms for measurement of protein levels of CEACAM-1 and MMP-2 using kits from R&D Systems (Wiesbaden, Germany). ELISA and quantitative analyses were performed according to the manufacturer’s protocol. The Mann-Whitney test (Statview software version 5.0) was performed to check for differences in plasma concentrations between the genotypes of the indicated SNPs, *P* < 0.05 considered significant. The red lines indicate the median of the plasma concentrations

Plasma concentrations of the proteins encoded by the genes associated with LE development were measured to evaluate the functional phenotypes. CEACAM-1 and MMP-2 were measured using commercially available kits to compare the plasma levels between LE patients and infected controls. The plasma levels of CEACAM-1 were significantly elevated in LE patients (*P* < 0.02, Fig. 1a). MMP-2 protein concentration was also significantly higher in the LE patients (*P* = 0.025, Fig. 1b).

**Fig. 2:**
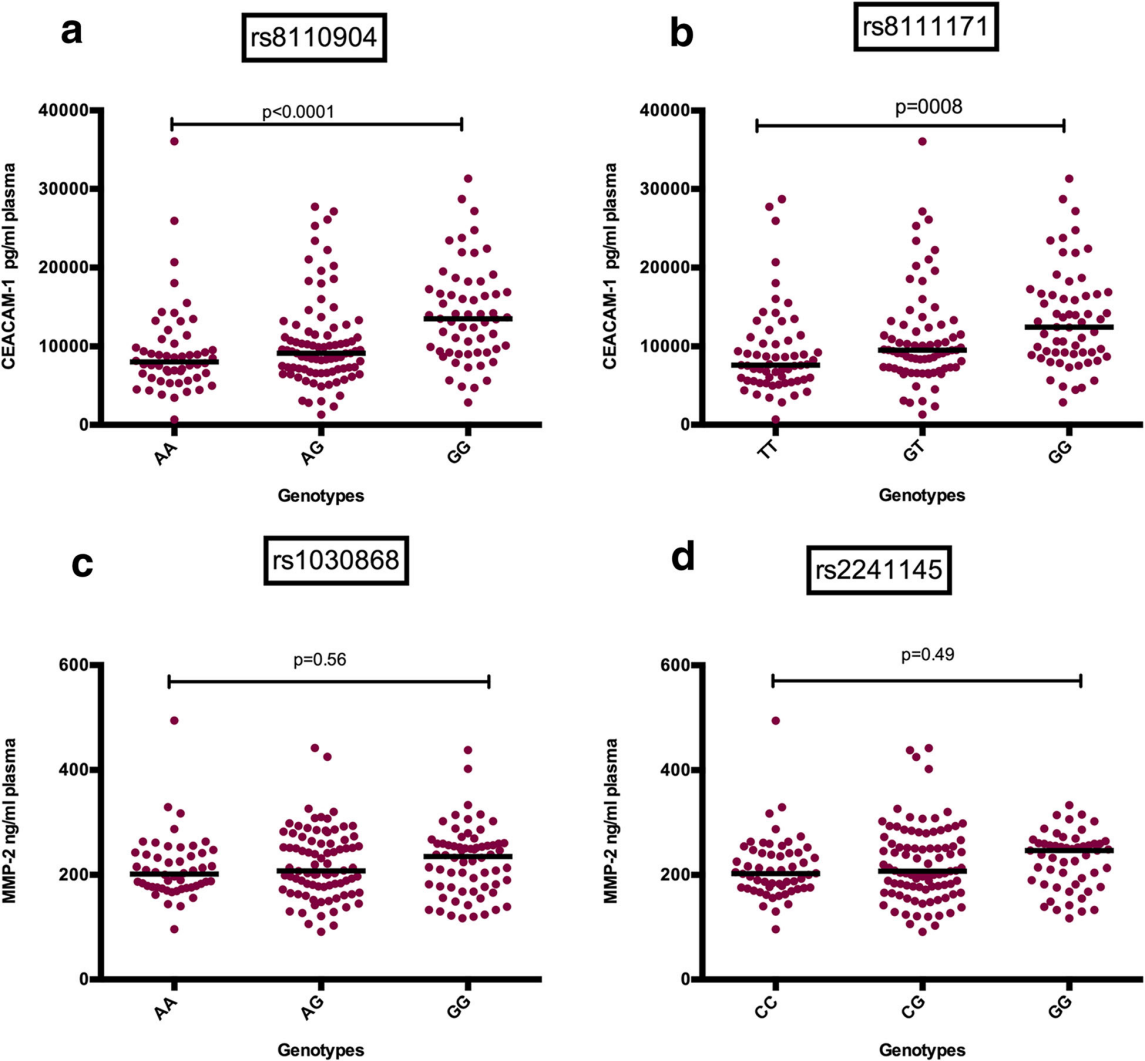
Functional characterization of associated SNPs in CEACAM-1 rs8110904, CEACAM-1 rs8111171, MMP-2 rs1030868, and MMP-2 rs2241145. **a** Plasma samples from patients with CEACAM-1 rs8110904 genotypes AA (*n* = 54), AG (*n* = 87), and GG (*n* = 59) were analyzed. **b** Plasma samples from patients with CEACAM-1 rs8111171 genotypes TT (*n* = 61), GT (*n* = 77), and GG (*n* = 61) were analyzed. The GG genotype in both SNPs had significantly higher plasma concentrations of CEACAM-1. **c** Plasma samples from patients with MMP-2 rs1030868 genotypes AA (*n* = 50), AG (*n* = 84), and GG (*n* = 66) were analyzed. **d** Plasma samples from patients with MMP-2 rs2241145 genotypes CC (*n* = 56), CG (*n* = 91), and GG (*n* = 53) were analyzed. No significant difference in the plasma levels was seen in the genotypes of either MMP-2 SNP. EDTA Plasma was collected from 101 LE patients and 99 infected patients without disease symptoms for measurement of protein levels of CEACAM-1 and MMP-2. ELISA and quantitative analyses were performed according to the R&D Systems (Wiesbaden, Germany) protocols. The Mann-Whitney test (Statview software version 5.0) was performed to check for differences in plasma concentrations between the genotypes of the indicated SNPs, *P* < 0.05 considered significant. The black lines indicate the median of the plasma concentrations

To functionally characterize the genotypes, plasma levels of CEACAM-1 and MMP-2 were correlated with the respective genotypes. CEACAM-1 plasma level was higher in people with the GG genotype in both rs8110904 and rs8111171 SNPs (Fig. 2). Plasma levels of MMP-2 did not correlate with any of the SNP genotypes in this population.

### Pathway interaction of lymphedema associated genes

**Fig. 3:**
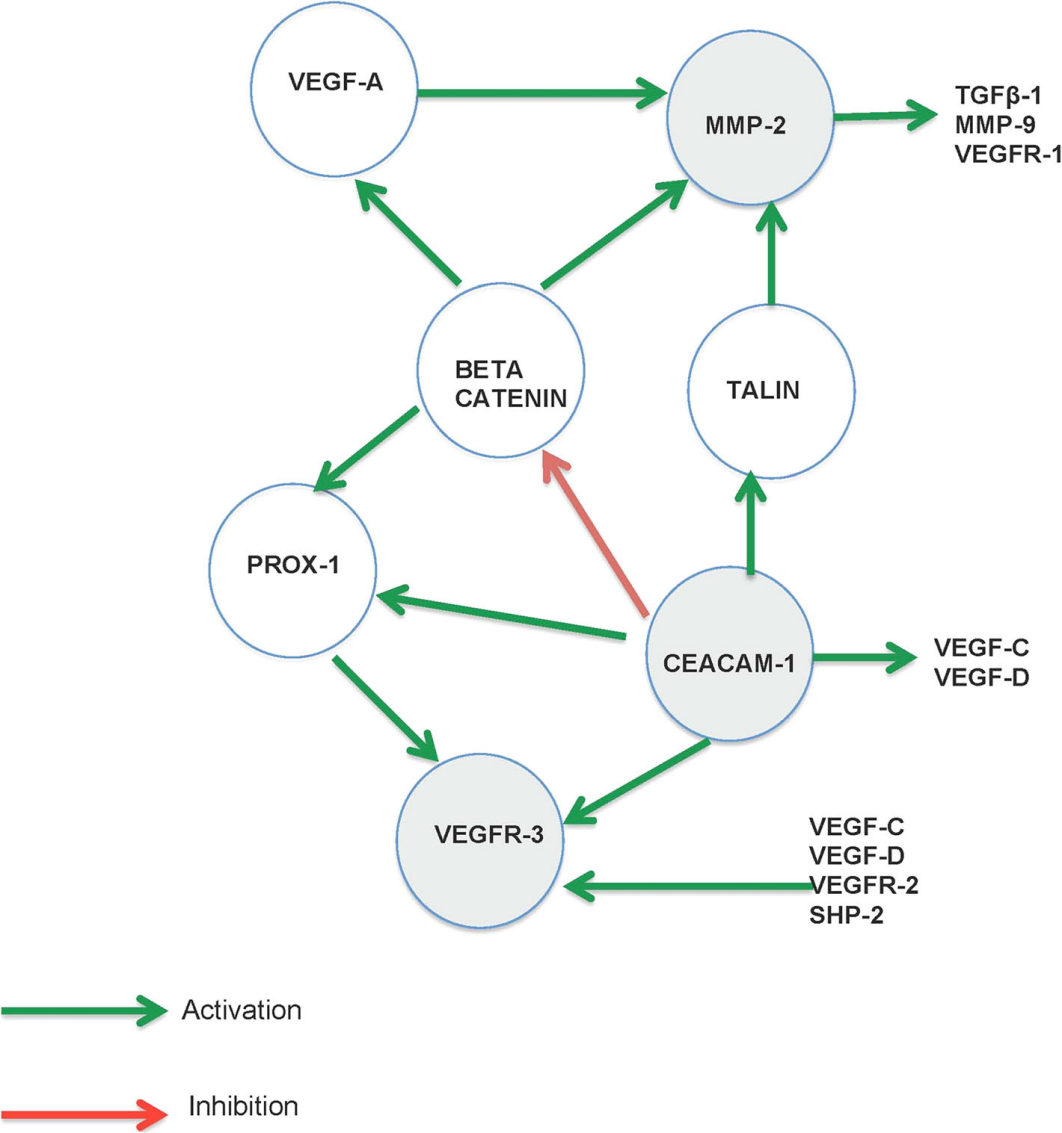
Pathway interaction of genes associated with lymphedema. Gene interactions were determined using MetaCore™software (GeneGo Inc., www.genego.com). All genes within a distance of two steps are indicated. Red arrows indicate a negative interaction (e.g., inhibition, down-regulation) while green arrows indicate a positive interaction (e.g., activation, up-regulation). Genes with gray backgrounds are genes significantly associated with lymphedema in this study

The MetaCore™ software package was used to analyze the genotyped genes for pathways of protein interaction. Because of the candidate gene approach of this case-control study, it is not surprising that the associated genes are in the angiogenesis pathway. Nevertheless, candidates for further study for their role in LE development are identified by this analysis. The three genes (gray circles) with SNPs associated with filarial LE interact with each other (CEACAM-1 and VEGFR-3) and 11 proteins in the angiogenesis pathway (Fig. 3). CEACAM-1 is predicted to up-regulate VEGFR-3 expression directly and up-regulates PROX-1, VEGF-C, and VEGF-D, which also up-regulate VEGFR-3. CEACAM-1 can also up-regulate MMP-2 via the up-regulation of TALIN. It down-regulates beta-catenin, a protein that up-regulates MMP-2 directly and also indirectly via up-regulation of VEFG-A, and up-regulates VEGFR-3 indirectly via PROX-1. MMP-2 is predicted to up-regulate TGF-β1, MMP-9, and VEGFR-1.

## Discussion

Pathological effects of lymphatic filariasis such as LE and hydrocele are observed in a fraction of the individuals in endemic areas even though up to 80% may be infected with *W. bancrofti* [5, 36]. LE, the most debilitating pathology, occurs in ~ 7% of the endemic population [6, 8].

Different studies have been undertaken to unravel the genetic basis of this heterogeneity, but they concentrated on infection and hydrocele development [24, 28]. Filarial LE is the single largest cause of secondary lymphedema [37], an inflammatory disease resulting from the destruction of the lymphatic vessel with associated fibrosis as a result of the presence and death of the adult filarial worms, larval death, and the release of *Wolbachia* endosymbionts [38]. Since the immune response of the host plays an integral role in disease etiology by inducing the expression of particular genes [38], host immunogenetics was exploited in this study to answer the question as to which SNPs could be causative variants in filarial LE development.

Five SNPs in three genes were identified to be associated with LE. The associations did not withstand correction for multiple testing, which is probably attributable to the low sample size and/or the small contributing effects of the SNPs on the disease.

Carcinoembryonic antigen-related cell adhesion molecule 1 (CEACAM-1) is a type 1 transmembrane protein involved in cell-to-cell adhesion [39]. It has been shown to be a potent stimulator of vascular endothelial growth factor (VEGF) mediated angiogenesis [40, 41]. It also stimulates microvascular endothelial cell growth in the presence of VEGF [40]. However, the overexpression of CEACAM-1 is associated with cancers such as thyroid cancer, gastric cancer, and metastasizing malignant melanomas [42].

Two SNPs in the CEACAM-1 gene (rs8110904 and rs8111171) were associated with LE development. The frequencies for the minor alleles A and T are consistent with the values reported for rs8111171 and rs8110904 from the Yorubian population (rs8110904 G = 57% A = 43%, rs8111171 G = 56% T = 44%) using 120 and 48 participants, respectively [43]. The minor allele A in rs8110904 was higher in the cases than in the controls, and patients with the A allele had an odds ratio of 1.2 (CI 0.99–1.49). The minor T allele frequency in rs8111171 was also higher in the cases than in the controls with a 1.3-fold risk (CI 1.03–1.56) of developing LE. The significant haplotype association of the “protective” haplotype GG (*P* = 0.0256) and haplotype of the case-associated alleles AT (*P* = 0.046) supports a role for this gene in disease development.

CEACAM-1 is up-regulated in some cancers such as thyroid and gastric cancers. The initial metastasis of these cancers is through the lymphatic vessels to the regional lymph nodes similar to the pathogenesis of LE which mainly occurs after dilation of the lymphatic vessel with associated fibrosis [44]. Multicellular activities such as angiogenesis have been attributed to encoded proteins of CEACAM-1. The serum of CEACAM-1 served as a useful indicator for the presence of pancreatic cancer [45]. CEACAM-1 is a potent inducer of VEGFs. The receptor 3 of VEGF-C and D has been associated with LE, and its plasma was found to be elevated in LE [27]. If CEACAM-1 stimulates VEGFs and the plasma levels of the receptors influence LE then higher plasma levels of CEACAM-1 in LE could play a role in the development of the disease. The significant increase in plasma protein concentration of CEACAM-1 gene in LE patients (Fig. 1a) correlates with the genotype and is an indication that CEACAM-1 might play a role in LE development. The plasma protein level was also observed to be higher with the GG genotype compared with the other genotypes indicating that the genotype GG directly or indirectly impacts the expression of CEACAM-1 plasma proteins and could be a variant for LE development.

Matrix metalloprotease-2 (MMP2) is a known angiogenic factor whose activity involves the breakdown of extracellular matrix in physiological processes such as embryonic development, reproduction, and tissue remodeling [46]. Mutations in the MMP-2 gene lead to a number of disease processes, such as arthritis and metastasis, tumor growth vascular aneurysmal disease development, Winchester syndrome, and nodulosis-arthropathy-osteolysis (NAO) syndrome [47–50].

The significant association of MMP-2 SNPs rs1030868 (*P* = 0.0094) and rs2241145 (*P* = 0.0116) is an indication that this gene might be involved in LE development. Patients with rs1030868 (minor allele A) and rs2241145 (minor allele C) SNPs have a 1.3-fold (CI 1.07–1.58 and CI 1.06–1.57, respectively) risk of developing LE than those who do not have these alleles (Table 2). In National Center for Biotechnology Information database of single nucleotide polymorphism (NCBI dbSNP), these minor alleles have a frequency of C = 52% (rs2241145) and A = 42% (rs1030868) in the Yorubian population (*n* = 120) and are similar to the values calculated from our larger sample size [43]. These SNPs have also been found to be associated with development of lacunar stroke [51], and higher levels of MMP-2 protein and activity have been described. The authors hypothesize that more MMP-2 alters and remodels the extracellular matrix around the vessels that contribute to the development of edema [52, 53], a hypothesis supported by the finding that MMP-2 also disrupts tight junctions [54]. Thus, extravasation of fluid occurs and contributes to stroke. A similar phenomenon is seen in LE development in which the lymph vessels dilate, reducing lymph flow. With destruction/remodeling of the vessel architecture, here hypothesized to be in part caused by MMP-2, lymph fluid enters the surrounding tissue causing lymphedema. The affected limb then enlarges progressively due to fibroadipose deposition [7].

MMP-2 mRNA levels are known to be higher in lymphedematous specimens compared to non-lymphedematous specimens of progenitor cells [7], and a blockage or down-regulation of this gene leads to reduced lymphangiogenesis [55]. Therefore, the significant increase in the plasma concentration of MMP-2 in LE patients (Fig. 1b) is an indication that this gene might have a role in the LE development. Even though the plasma concentration was evenly distributed among the genotypes (Fig. 2c, d), the identified associations of these intronic SNPs seem to account for another genetic effect which is independent of the plasma level. Thus, these intronic SNPs may act as proxy markers for another, yet to be identified, functional SNP in this chromosomal region.

Tetracycline and its derivatives have been shown to profoundly inhibit mammalian MMPs by a mechanism that is independent of their antimicrobial activity, thereby reducing excessive degradation or remodeling at the healing enthesis after rotator cuff repair [56, 57]. It has been shown by Debrah et al. [27, 58] that doxycycline improves the condition of disease symptoms of LF patients with early stages of LE. However, the mechanism of action was not clear. This study supports that the effect might be a direct effect on MMP-2 and explains why doxycycline is able to ameliorate LE pathology even though most LE patients do not have active infections. Additionally, pathway analysis with MetaCore shows that MMP-2 positively influences the expression of VEGFs, and therefore, inhibition of MMP-2 may result in additive or synergistic effects with other factors in this pathway.

VEGFR-3, a tyrosine-protein kinase, emerged as one of the genes associated with LE development in this study. A single marker association was found between the VEGFR-3 SNP (rs75614493) in the exon region of chromosome 5 and LE development (Table 2, *P* = 0.034). There was no patient homozygous TT in the study population even though 1 and 2% of patients carried the T allele in the cases and control groups, respectively. The allelic distribution from this study is consistent with earlier work done in sub-Saharan Africa (Yorubian population) involving 118 patients (NCBI dbSNP). The allelic frequency of the study was 97.5% for the C allele and 2.5% for the T allele [59].

From this present study, the C allele was significantly more frequent in the cases compared to the controls with an odds ratio of 3.4 (CI 1.02–11.29). Even though the genotype frequencies are 99 and 96% in cases vs controls, a statistical difference was observed and a significant difference in haplotype frequencies between rs75614493 and rs3587489 was also observed in the TT haplotype with one degree of freedom.

VEGFR-3 is restricted largely to the lymphatic endothelium and acts as a cell surface receptor for VEGF-C and VEGF-D [60]. The other two receptors of VEGFs that have been identified, VEGFR-1 and VEGFR-2, are expressed mainly in the blood vascular endothelium [61]. Studies on the molecular mechanisms controlling the lymphatic vessels have shown that vascular endothelial growth factors C and D specifically control lymphangiogenesis in humans by activating the VEGF receptor-3 (VEGFR-3) [62, 63] [61, 64]. VEGFR-3 has also been linked to human hereditary LE [65].

VEGFs and VEGFR3 are needed for the development of lymphatic vessels. However, their over-production leads to lymphatic dilation and LE development [66]. In animal models, overexpression of VEGF-C in the skin of transgenic mice resulted in lymphatic endothelial proliferation and dilation of lymph vessels [64] with a resemblance to lymphatics infected with filarial parasites. These transgenic mice then developed a lymphedema-like phenotype characterized by swelling of feet, edema, and dermal fibrosis [67], similar to what is observed in humans.

Several studies by Debrah et al. [27, 28, 58], involving our study participants, showed that plasma levels of VEGFs and a VEGF soluble receptor, sVEGFR-3, are significantly elevated in patients infected with filarial worms, and a correlation was found between sVEGFR-3, lymphatic dilation, and pathology development. Targeting the filarial worms by doxycycline reduced the levels of VEGFs/sVEGFR-3, with amelioration of dilated supratesticular lymphatic vessels and reduction in LE and hydrocele stages. A mechanism that could be due to the non-antimicrobial activity of tetracyclines. The fact that the VEGF and sVEGFR-3 reduction preceded the improvement of pathology indicates a possible causal interaction between lymphangiogenic factors and lymphatic pathology, rather than only a coincidence or an epiphenomenon.

Pathway interaction of associated genes was done using the MetaCore software package [68]. Genes in the angiogenic pathway were shown to be involved in a complex relationship (Fig. 3). Genes in gray background had SNPs that were directly associated with LE in this study (MMP-2, CEACAM-1, and VEGFR-3). However, during pathway analysis, other genes were found to either activate or inhibit those genes that were found to be directly associated with LE.

CEACAM-1, MMP-2, and VEGFR-3 genes are directly found to be involved in LE development. The pathway interaction of LE associated genes provides information on the involvement of other genes and probably other SNPs in the development of LE. CEACAM-1 is known to be involved in angiogenesis, and its overexpression in human dermal microvascular endothelial cells (HDMEC) leads to an up-regulation of VEGF-C, VEGF-D, and VEGFR-3 [39, 69]. The down-regulation of CEACAM-1 results in deregulation of beta-catenin, which is known to be associated with malignant transformation [70, 71]. TALIN interacts with CEACAM-1 and increases its activity [72].

Prospero homeobox protein-1 (PROX-1) activates VEGFR-3 with a subsequent increase in receptor expression [73]. Activation of PROX-1 is positively regulated by beta-catenin signaling [74]. VEGF-A is essential for cancer neovascularization and cancer invasion by promoting endothelial mitogenesis and permeability. However, the overexpression is known to increase MMP-2 levels in glioblastoma [75]. At the same time, MMP-2 up-regulates TGF beta-1, MMP-9, and VEGFR-1 (Fig. 3). The interaction of these genes can, therefore, be said to contribute to the disease.

This study is the first to determine the genotype frequencies of CEACAM-1 and MMP-2 in our study population. We have gone a further step to confirm the involvement of lymphangiogenic/angiogenic factors in the development of pathology of LF. Our pathway analysis also supports the assertion that LE due to *W. bancrofti* infection is a complex disease and not caused by a single genetic factor.

## Conclusion

SNPs in the angiogenic and lymphangiogenic pathway contribute to the development of filarial LE. The genes whose SNPs were found to be associated with LE (CEACAM-1, MMP-2, and VEGFR-3) have an influence in the vascular endothelial growth factors either directly or indirectly, supporting the fact that VEGFs are major functional proteins in filarial LE development and that the identified angiogenic/lymphangiogenic factors function through influencing the VEGFs. The direct activity of MMP-2 on the extracellular matrix which results in the progressive damage of vascular walls in vascular diseases when elevated could also have direct influence in the development of LE. The outcomes of this study are important in the diagnosis of the disease as well as the development of future vaccine. Although the associations were not as strong as anticipated, they underscore the fact that LE is a complex disease caused by multiple genetic markers. This complex interaction of genes therefore calls for a first-stage genome-wide association study (GWAS) to identify all genes associated with LE development that could serve as markers for diagnosis of the disease, and identify pathways that could be targeted by chemotherapeutics to prevent/reduce lymphedema, providing amelioration of disease to the thousands of people with LE.

The 1000 Genome haplotypes is a valuable resource to infer information on further SNPs via genotype imputation. Unfortunately, the number of SNPs per linkage disequilibrium/gene region available in our study was not sufficient to enable the application of the IMPUTE2 software. In our ongoing GWAS study with a larger sample size, we hope to be able to achieve denser genotyping to be able to make use of public resources like 1000 Genomes or the haplotype reference panel (http://www.haplotype-reference-consortium.org/).

## Methods

### Study design

The study participants were selected from the Nzema East and Ahanta West districts in the Western Region of Ghana, which are the LF endemic districts in Ghana.

Participants numbering 967—comprising 285 lymphedema patients (Cases) and 682 infected patients without pathology (controls) were enrolled into the study. All volunteers included in the study underwent finger prick blood collection at night for assessment and quantification of microfilariae (Mf) in the peripheral blood. Circulating filarial antigen (CFA) test to identify infected patients who did not have Mf was also done. The procedures for microfilaria and CFA determination were done as described [27, 76]. LE patients were examined separately by a clinician conversant with the symptoms of LF, and the staging was done according to Dreyer et al. [32].

### Genotyping

#### DNA extraction

In the field lab, volunteer’s blood was mixed with equal volumes of 8 M urea for preservation at ambient temperature. The Chemagen platform (Chemagen Biopolymer-Technologies AG, Baesweiler, Germany) in Bonn was used for the DNA extraction as per kit instructions. After the genomic DNA was isolated, a DNA stock concentration of 100 ng/ml was diluted to 15 ng/μl working concentration with Tris-EDTA buffer. Quality-checked DNA samples with an A260/A280 ratio between 1.7 and 2.0 were pipetted into aliquots of 2 μl (30 ng DNA) per well in a 384-well plate for genotyping.

#### SNP genotyping

To investigate several candidate genes conferring susceptibility or protection to LE, the SNP databases National Center for Biotechnology Information (NCBI) and Online Mendelian Inheritance in Man (OMIM) were used [77, 78]. These databases provide information on genetic variations. SNP Annotation and Proxy Search (SNAP) (http://www.broadinstitute.org) was also used to find proxy SNPs based on linkage disequilibrium, physical distance, and/or membership in selected commercial genotyping arrays. Pair-wise linkage disequilibrium was pre-calculated based on phased genotype data from the International HapMap Project (www.hapmap.org), which has since been replaced by the 1000 Genomes Project (www.1000genomes.org).

In all, 64 genes of interest known to have a role in inflammation, angiogenesis/lymphangiogenesis, extravasation of fluid, and also in other mechanisms, such as cell differentiation during tumorigenesis, were selected. A total of 131 functional variants from the 64 genes were successfully genotyped and analyzed (Additional file 2: Table S1). Genotyping was done in multiplex reactions using the MassARRAY (Sequenom Inc., San Diego, USA) platform. Identified monoallelic SNPs and SNPs with genotyping call rates of < 95% were excluded from the analysis.

### Determination of plasma levels of angiogenic/lymphangiogenic molecules

Plasma concentrations of the angiogenic/lymphangiogenic molecules associated SNPs were assessed as a measure of the functional phenotypes in the genotyped samples. Blood from cases and controls was taken with ethylenediaminetetraacetic acid (EDTA) tubes, and plasma was collected for measurement of protein levels of CEACAM-1 and MMP-2 using commercially available kits (R&D Systems, Wiesbaden, Germany) according to the manufacturer’s protocol. ELISA plates were read with a Wallac VICTOR^2^ 1420 (PerkinElmer Inc., Waltham MA, USA) at 450 nm and corrected with a second read at 540 nm. A standard curve was created for each plate using a four-parameter logistic (4-PL) curve fit. Only plates with a standard curve r^2^ > 0.99 were evaluated.

### Statistical analyses

FamHap version 19 software was used for single marker analysis as well as haplotype analysis of association of the SNPs with cases or controls [79]. Genotype and haplotype frequencies were summarized as percentages. Statistical significance for the single marker SNP analysis was calculated using the Cochrane-Armitage test for trend with P_ATT_ ≤ 0.05 considered significant. The Armitage test is less influenced by deviation from Hardy-Weinberg equilibrium (HWE), and the result obtained is valid and acceptable even when a group is not in HWE [35]. Genotype-specific risks were estimated as odds ratios (ORs) with 95% confidence intervals (CIs). Analysis of dominant or recessive association was done using the DeFinetti formula at ihg.gsf.de/cgi-bin/hw/hwa1.pl.

Haplotype analyses calculated an omnibus statistic using 200,000 simulations of the case-control data correcting for multiple testing (global *P* value). MetaCore™ software (GeneGo Inc., St. Joseph, MI, USA) was used to test for SNP interaction and network analysis [68]. Unpaired *t* test with GraphPad Prism version 6 (La Jolla, California, USA, www.graphpad.com) software was used for comparing the differences in the plasma concentration of the samples and for plotting the graphs from data generated. *P* ≤ 0.05 were considered statistically significant.

## Additional files

Additional file 1: List of genotyped single nucleotide polymorphisms (DOCX 35 kb)

Additional file 2: Table S1. Model of association for filarial lymphedema risk alleles (DOCX 13 kb)

## Abbreviations

CEACAM-1: Carcinoembryonic antigen-related cell adhesion molecule-1
CHIT-1: Chitinase-1
CTLA-4: Cytotoxic T-lymphocyte-associated protein 4
EDTA: Ethylenediaminetetraacetic acid
ELISA: Enzyme-linked immunosorbent assay
IL-4: Interleukin-10
LE: Lymphedema
LF: Lymphatic filariasis
MBL-2: Mannose-binding lectin-2
MHC: Major histocompatibility complex
MMP-2: Matrix metalloprotease-2
PROX-1: Prospero homeobox protein-1
SNP: Single nucleotide polymorphism
TGFβ-1: Transforming growth factor-β-1
TNF-α: tumor necrosis factor-alpha
VEGFR-3: Vascular endothelial growth factor receptor-3
