# Supplementary material for: Single nucleotide polymorphisms in the angiogenic and lymphangiogenic pathways are associated with lymphedema caused by Wuchereria bancrofti
Source: Hum Genomics. 2017 Nov 9;11:26. doi: 10.1186/s40246-017-0121-7 (PMC5679374; doi:10.1186/s40246-017-0121-7)
Supplement: Supplementary file 1 — List of genotyped single nucleotide polymorphisms (DOCX 35 kb) [file 40246_2017_121_MOESM1_ESM.docx]

Additional file1: List of genotyped single nucleotide polymorphisms

|  | **Gene name** | **Rs number** | **Amino Acid Change** | | **Gene Region** | |
| --- | --- | --- | --- | --- | --- | --- |
| 1 | TLR 9 | 352139 | None | | Promoter | |
| 2 | TLR 9 | 5743836 | None | | Promoter | |
| 3 | TLR 2 | 3804099 | None | | Coding Exon | |
| 4 | TLR 2 | 3804100 | None | | Coding Exon | |
| 5 | TLR 2 | 893629 | None | | Promoter | |
| 6 | TLR 2 | 4585282 | None | | Intron | |
| 7 | TLR 2 | 7656411 | None | | Near-Gene-3* | |
| 8 | TLR 4 | 5030725 | None | | Intron | |
| 9 | IL-5 | 2069807 | None | | Promoter | |
| 10 | IL-5 | 2069812 | None | | Promoter | |
| 11 | IL-5 | 2069818 | 128 Thr/Thr | | Coding Exon | |
| 12 | IL-23R | 7530511 | 310 Leu/Pro | | Coding Exon | |
| 13 | IL-23R | 1884444 | 3 Gln/His | | Coding Exon | |
| 14 | NFKB INHIBITOR ALPHA | 28933100 | 32 Ser/Ile | | Coding Exon | |
| 15 | NFKB INHIBITOR ALPHA | 696 | None | | 3' Utr | |
| 16 | NFKB INHIBITOR ALPHA | 2233406 | None | | Promoter | |
| 17 | NFKB INHIBITOR ALPHA | 3138053 | None | | Promoter | |
| 18 | MYD88 (ACAA1) | 4988453 | None | | Promoter | |
| 19 | ENDOSTATIN (COL18 A1) | 12483377 | 1257Asp/Asn | | Coding Exon | |
| 20 | ENDOSTATIN (COL18 A1) | 62000965 | 259 Arg/Leu | | Coding Exon | |
| 21 | ENDOSTATIN (COL18 A1) | 76547444 | 517 Gly/Ser | | Coding Exon | |
| 22 | ENDOSTATIN (COL18 A1) | 62000962 | 841 Val/Ile | | Coding Exon | |
| 23 | I-CAM | 5498 | 469 Lys/Glu | | Coding Exon | |
| 24 | I-CAM | 5491 | 56 lys/Met | | Coding Exon | |
| 25 | V-CAM | 3783615 | 624 Ile/Leu | | Coding Exon | |
| 26 | V-CAM | 1041163 | None | | Promoter | |
| 27 | V-CAM | 3176879 | 644 lys/Lys | | Coding Exon | |
| 28 | VEGF-C | 7664413 | None | | Intron (Boundary) | |
| 29 | VEGF-C | 41278571 | 61 Arg/Glu | | Coding Exon | |
| 30 | VEGF-C | 55728985 | 47 Glu/Val | | Coding Exon | |
| 31 | VEGF-C | 6838834 | None | | Intron | |
| 32 | VEGF-D | 5980152 | None | | Promoter | |
| 33 | VEGF-D | 4830943 | None | | Promoter | |
| 34 | ANG-2 | 2515475 | None | | Intron | |
| 35 | ANGPT 2 | 7814961 | 32 Lys/Asn | | Coding Exon | |
| 36 | BFGF | 1449683 | None | | Coding Exon | |
| 37 | VEGF-R1 | 78147150 | 861 Lys/Glu | | Coding Exon | |
| 38 | VEGF-R1 | 35832528 | 982 Glu/Ala | | Coding Exon | |
| 39 | VEGF-R3 | 35874891 | 527 Asn/Ser | | Coding Exon | |
| 40 | VEGF-R3 | 75614493 | 1154 Gly/Arg | | Coding Exon | |
| 41 | TIMP-1 | 61756234 | 65 Met/Lle | | Coding Exon | |
| 42 | TIMP-2 | 2277698 | 101 Ser/Ser | | Coding Exon | |
| 43 | CEACAM-1 | 8111171 | 35 Gln/Glu | | Coding Exon | |
| 44 | CEACAM-1 | 8110904 | 83 Gln/Lys | | Coding Exon | |
| 45 | LYVE-1 | 746164 | None | | Intron | |
| 46 | LYVE-1 | 16907980 | 214 Thr/Lle | | Coding Exon | |
| 47 | PROX-1 | 77561623 | 150 Ser/Arg | | Coding Exon | |
| 48 | PROX-1 | 78142452 | 479 Thr/Lle | | Coding Exon | |
| 49 | MMP-2 | 11643630 | None | | Promoter | |
| 50 | MMP-2 | 2241145 | None | | Intron | |
| 51 | MMP-2 | 1992116 | None | | Intron | |
| 52 | MMP-2 | 1030868 | None | | Intron Boundary | |
| 53 | MMP-9 | 17576 | 279 Gln/Arg | | Coding Exon | |
| 54 | TBK-1 | 17853341 | 570 Lys/Gln | | Coding Exon | |
| 55 | TBK-1 | 35635889 | 464 Val/Ala | | Coding Exon | |
| 56 | GJA4 | 1764391 | 319 Pro/Ser | | Coding Exon | |
| 57 | CD 36 | 1334512 | None | | Promoter | |
| 58 | CD 36 | 3211938 | 325 Tyr/ Ter | | Coding Exon | |
| 59 | ANGPTL4 | 1044250 | 266 Thr/Met | | Coding Exon | |
| 60 | CTLA-4 | 3087243 | None | | Near-Gene-3* | |
| 61 | OLFML3  (OLFACTOMEDIN-LIKE 3) | 35317518 | 187 Arg/Ser | | Coding Exon | |
| 62 | OLFML3  (OLFACTOMEDIN-LIKE 3) | 11553080 | 46 Aspglu | | Coding Exon | |
| 63 | NOD2/CARD15 | 3135500 | None | | 3'utr | |
| 64 | NOD2/CARD15 | 5743291 | 955 Val/Lle | | Coding Exon | |
| 65 | NOD2/CARD15 | 1000331 | None | | intron | |
| 66 | NOD 1 | 2075820 | 266 Glu/Gln | | Coding Exon | |
| 67 | NOD 1 | 736781 | None | | Intron | |
| 68 | ATG 16 L1 | 2241880 | 197thr/Ala | | Coding Exon | |
| 69 | IL-23R | 7517847 | None | | Intron | |
| 70 | 5-LO | 2228065 | None | | Coding Exon | |
| 71 | CAVEOLIN-1 | 4730751 | None | | Intron | |
| 72 | CAVEOLIN-1 | 926198 | None | | Intron | |
| 73 | CAVEOLIN-1 | 4730748 | None | | Intron | |
| 74 | MBL-2 | 7095891 | None | | Promoter | |
| 75 | MBL-2 | 10824792 | None | | 3' Utr | |
| 76 | FGF-2 | 308447 | None | | Promoter | |
| 77 | FGF-2 | 303379 | None | | Intron | |
| 78 | HGF | 74657718 | None | | Intron | |
| 79 | IGF-1 | 2946834 | None | | Downstream | |
| 80 | IGF-1 | 7136446 | None | | Intron | |
| 81 | DESMOPLAKIN | 2076299 | 1512 Tyr/Cys | | Coding Exon | |
| 82 | DESMOPLAKIN | 6929069 | 1738 Arg/Gln | | Coding Exon | |
| 83 | BETA-CHEMOKINE RECEPTOR D6 | 2228468 | 373 Tyr/Ser | | Coding Exon | |
| 84 | BETA-CHEMOKINE RECEPTOR D6 | 6779520 | 311 Leu/Val | | Coding Exon | |
| 85 | COL1A1 | 2075555 | None | | Intron Boundary | |
| 86 | COL1A1 | 2269336 | None | | Near-Gene-5^#^ | |
| 87 | COL1A2 | 42524 | 549 Pro/Ala | | Coding Exon | |
| 88 | COL1A2 | 1034620 | None | | Downstream | |
| 89 | CPLA2 ALPHA | 3820185 | None | | Intron | |
| 90 | CPLA2 ALPHA | 12749354 | None | | Intron | |
| 91 | GATA 2 | 2335052 | 164 Ala/Thr | | Coding Exon | |
| 92 | GATA 2 | 3803 | None | | 3'utr | |
| 93 | GATA 2 | 2713604 | None | | Intron | |
| 94 | GATA 3 | 570613 | None | | Intron | |
| 95 | GATA 3 | 3803604 | None | | Intron | |
| 96 | IL-10 | 1800896 | None | | Promoter | |
| 97 | IL-10 | 1800871 | None | | Promoter | |
| 98 | IL-10 | 1800872 | None | | Promoter | |
| 99 | IL-6R | 2228145 | 358 Asp/Ala | | Coding Exon | |
| 100 | VEGFR-2 | 2305948 | 297 Val/Ile | | Coding Exon | |
| 101 | VEGFR-2 | 1870377 | 472 His/Gln | | Coding Exon | |
| 102 | VEGFR-2 | 1531289 | None | | Intron Boundary | |
| 103 | TLR-6 | 13102250 | | 105 Trp/Leu | | Coding exon |
| 104 | TLR-6 | 5743810 | | 249 Pro/Ser | | Coding exon |
| 105 | TLR-6 | 5743813 | | 456 Tyr/His | | Coding exon |
| 106 | IL-4 | 2243250 | | None | | Promoter |
| 107 | IL-13 | 20541 | | 110Arg/Gln | | Coding exon |
| 108 | IL-13 | 2069739 | | None | | Promoter |
| 109 | IL-13 | 1800925 | | None | | Promoter |
| 110 | IL-13 | 2069743 | | None | | Promoter |
| 111 | ENDOTHELIN-1 | 5370 | | 198 Lys/Asn | | Coding exon |
| 112 | ENDOTHELIN-1 | 1800541 | | None | | Promoter |
| 113 | ENDOTHELIN-1 | 35104761 | | 82Tyr/Val | | Frameshift |
| 114 | TNFA | 1800629 | | None | | Near gene 5^#^ |
| 115 | TNFA | 1799964 | | None | | Near gene 5^#^ |
| 116 | TNFA | 1799724 | | None | | Near gene 5^#^ |
| 117 | VEGF-A | 833061 | | None | | Promoter |
| 118 | VEGF-A | 2010963 | | None | | 5’utr |
| 119 | VEGF-A | 1570360 | | None | | Promoter |
| 120 | IL-17F | 763780 | | 161 His/Arg | | Coding exon |
| 121 | IL-17A | 2275913 | | None | | Promoter |
| 122 | IL-6 | 1800796 | | None | | Promoter |
| 123 | IRF5 | 4728142 | | None | | Promoter |
| 124 | IRF5 | 3807306 | | None | | Promoter |
| 125 | TLR4 | 4986790 | | 229 Arg/Gly | | Coding exon |
| 126 | IL-18 | 187238 | | None | | Promoter |
| 127 | IL-18 | 1946518 | | None | | Promoter |
| 128 | IL-18 | 360718 | | None | | 5’utr |
| 129 | IL-18 | 1946519 | | None | | Promoter |
| 130 | IFN-G | 2069720 | | None | | Intron |
| 131 | IFN-G | 1861494 | | None | | Intron |
| 132 | IFN-G | 2069707 | | None | | Promoter |
| 133 | DECORIN | 17018909 | | 64 Leu/Val | | Coding exon |
| 134 | DECORIN | 3138268 | | 268 Met/Thr | | Coding exon |
| 135 | DECORIN | 1803344 | | 273 Gln/ Glu | | Coding exon |
| 136 | DECORIN | 1803342 | | 299 Pro/ Leu | | Coding exon |
| 137 | EOSINOPHIL  CATIONIC PROTEIN | 2073342 | | 124Thr/Arg | | Coding exon |
| 138 | IL-4R | 1805010 | | 75 Lle/Leu | | Coding exon |
| 139 | IL-4R | 1049631 | | None | | 3’utr |
| 140 | IL-4R | 1805015 | | 503Ser/Pro | | Coding exon |
| 141 | TGF-BETA | 1800469 | | None | | Promoter |
| 142 | TGF-BETA | 1800468 | | None | | Promoter |
| 143 | TGF-BETA | 1800470 | | 10Leu/Pro | | Coding exon |
| 144 | TGF-BETA | 1800471 | | 25Arg/Pro | | Coding exon |
| 145 | FOXP3 | 55711326 | | 44 Ser/Thr | | Coding exon |
| 146 | FOXP3 | 17847095 | | 52Val/Gly | | Coding exon |
| 147 | FOXP3 | 2232369 | | 220Val/Ala | | Coding exon |

* SNP within 0.5kb 3' to a gene # SNP within 2kb 5' to a gene Highlighted yellow SNPs not genotyped
