# Supplementary material for: Single nucleotide polymorphisms in the angiogenic and lymphangiogenic pathways are associated with lymphedema caused by Wuchereria bancrofti
Source: Hum Genomics. 2017 Nov 9;11:26. doi: 10.1186/s40246-017-0121-7 (PMC5679374; doi:10.1186/s40246-017-0121-7)
Supplement: Supplementary file 2 — Model of association for filarial lymphedema risk alleles (DOCX 13 kb) [file 40246_2017_121_MOESM2_ESM.docx]

**Supplemental Table 1. Model of association for filarial lymphedema risk alleles**

| **Gene, risk allele**  **(dbSNP rs#)** | **Dominance model**  **P-value^a^** | **OR^b^**  **(95% CI)** | **Recessive model**  **P-value** | **OR**  **(95% CI)** |
| --- | --- | --- | --- | --- |
| CEACAM-1, A  (rs8110904) | **0.008^c^** | 1.5  (1.12-2.13) | 0.689 | 0.91  (0.56-1.47) |
| CEACAM-1, T  (rs8111171) | **0.007** | 1.57  (1.13-2.17) | 0.352 | 0.86  ( 0.62-1.18) |
| FLT-4/VEGFR-3, C  (rs75614493) | 0.034 | 3.4  (1.02-11.48) | n.a. | n.a. |
| MMP-2, A  (rs1030868) | 0.112 | 0.78  (0.58-1.06) | **0.006** | 1.61  (1.15-2.26) |
| MMP-2, C  (rs2241145) | 0.063 | 0.73  (0.53-1.02) | **0.022** | 1.43  (1.05-1.95) |

^a^ ihg.gsf.de/cgi-bin/hw/hwa1.pl

^b^ Odds Ratio with 95%confidence intervals for the risk allele

^c^ bold text indicates the inheritance model for the risk allele
